# Supporting validity: steps to contextualise applications for technology and assessment, for learning

**DOI:** 10.12688/mep.19688.1

**Published:** 2023-07-19

**Authors:** Sandra Kemp, Viktoria C. T. Goddard, Katharine Boursicot, Richard Fuller, Vishna Devi Nadarajah

**Affiliations:** 1 University of Wollongong, Wollongong, New South Wales, Australia; 2 University of Liverpool, Liverpool, England, UK; 3 Health Professional Assessment Consultancy, Singapore, Singapore; 4 Christie Education, Manchester, UK; 5 International Medical University, Kuala Lumpur, Malaysia

**Keywords:** validity, learning, technology, sustainability, equity, assessment

## Abstract

In the paper, the authors offer perspectives on the uses of technology and assessment, that support learning. The perspectives are viewed through validity (from the field of assessment) as a framework and they discuss four aspects of an interconnected technology, learning and assessment space that represent theory informed, authentic practice. The four are: 1) integrated coherence for learning, assessment and technology; 2) responsibilities for equity, diversity, inclusion and wellbeing; 3) sustainability; and 4) balancing resources in global contexts. The authors propose steps and considerations for medical and health professions educators who need to contextualise applications for technology, learning and assessment, for positive impact for learners, faculty, institutions and patient care.

## Introduction

For educators in the medical and health professions, the Ottawa consensus statements represent an expert view of evidence-informed best practice ( Issenberg, 2011). Two recent statements explored issues of assessment, technology and the impact on learners and learning: Performance Assessment ( Boursicot *et al*., 2021) and Technology in Assessment ( Fuller *et al*., 2022). Key messages from these consensus statements are briefly summarised in Table 1 below.

**Table 1.** Key messages from Consensus Statements.

Performance Assessment Consensus Statement	Technology in Assessment Consensus Statement
Trends in past 10 years relate to systems of assessment, validity standards for assessment, rater cognition, and feedback.	Important aspects relate to evaluation of readiness to adopt technology, application of assessment technology, and evaluation/dissemination.
OSCEs (Objective Structured Clinical Examinations) and WBAs (Workplace Based Assessments) are important methods of performance assessment, and have different purposes, with well-established quality criteria for design including: OSCEs: adequate sampling; marking schemes aligned with clinical thinking; standard setting and metrics; value of examiner diversity; test security through task design, and circuit design. WBAs: use narrative feedback, marking schemes to reflect clinical performance, system to encourage desirable learning behaviours, interpret data holistically, and be aware of the ‘failure to fail’ phenomenon.	When applying technology, an assessment ‘lifecycle’ approach should target five key foci: (1) advancing authenticity of assessment, (2) engaging learners with assessment, (3) enhancing design and scheduling, (4) optimising assessment delivery and recording learner achievement, and (5) tracking learner progress and faculty activity and thereby continuous assessment.
Performance assessment should: - drive desirable learning behaviours. - account for psychometric implications. - include narrative feedback for learning.	Technology should support longitudinal learning and be: - enabler of assessment with active engagement of learners and faculty. - planned with active involvement of learners.

A wide body of work in the fields of assessment and technology in assessment, including these two consensus statements, has contributed to deeper understanding of learners and learning, and both assessment *for* learning and assessment *of* learning. Educators and supervisors of students and trainees (in the paper we subsequently refer to these as ‘learners’) in the medical and health professions, can draw upon decades of academic work to understand learning from theoretical and authentic, practice base viewpoints. Work by the National Academies of Sciences, Engineering and Medicine (2018) has been instrumental in synthesising knowledge from the fields of education and learning sciences on how individuals learn, building on theories of learning ( Piaget, 1950; von Glasersfeld, 1995; Wertsch, 1985). From the fields of measurement and evaluation, assessors of learners are able to follow testing and assessment standards developed through work on validity and test validation, readily accessible through influential publications such as the *Standards of Educational and Psychological Testing* ( American Educational Research Association *et al*., 2014) and the work of Kane ( Clauser & Bunch, 2022; Kane, 2013). Concurrently, a substantial body of work supports the interface of these two areas through the design and application of assessment for learning ( Black *et al*., 2007; Boud, 2007; Wiliam, 2018).

The challenge for educators is the synthesis and application from these diverse fields: that is, how learning and assessment interfaces with technology use in a specific medical or health professional education programme, within a particular healthcare setting, and embedded in a particular country/jurisdiction. Further challenges arise from rapid and continual developments with emerging and adaptive technologies ( Shankar, 2022) and the current debates surrounding the opportunities and challenges of artificial intelligence ( Cotton *et al*., 2023).

In this paper, we draw upon validity (from the field of assessment) as a framework to discuss four aspects of an interconnected technology, learning and assessment space that are critical components of validity ‘evidence’ ( Cook *et al*., 2015). The four are: 1) coherence; 2) equity, diversity, inclusion and wellbeing; 3) sustainability; and 4) global resources. We propose steps and considerations for all health professions educators who need to contextualise applications for technology, learning and assessment, for positive impact.

### 1) Steps towards integrated coherence for learning, assessment and technology

A key aspect of validity is being attentive to coherence between learning and assessment. To extend the original work on ‘constructive alignment’( Biggs & Tang, 2011), technology is now integrated into curriculum, learning experiences, and assessment tasks. The challenge is adopting an approach to achieve coherence in this integration. Validity underpins the test validation and technical processes we need to adopt to ensure that the intended use of our assessment, the sources of validity evidence we generate, and the inferences we draw from assessment data can be justified ( Kane, 2013). In this way, validity provides a way of thinking about purpose of the learning and assessment activities and underpins evaluation of whether there is a desirable impact on learners and learning, as well as achievement. Key questions to ensure integrated coherence across learning, assessment and technology include:

a) How do we check curriculum and learning alignment, using assessment data?
b) How well do we provide actionable feedback to learners? (i.e. feedback from assessors that enables learners to take action to improve learning)
c) A need to look beyond simple competency-based education to ask, what are the outcomes of this coherence for learners, faculty, programmes and patients?

Technology affordances in these areas are increasing. For educators who need to check curriculum and learning alignment, technology now enables support of multi-dimensional curriculum-assessment blueprinting, to generate data related to performance on detailed, broad/specific aspects of curriculum and assessment. Contemporary technology allows learner assessment data to be interrogated at cohort level. Some examples are:

- assessment data indicates a cohort of learners performed well across examination items blueprinted to a learning outcome related to the topic of ‘prescribing’. The same cohort performs poorly on ‘management of the rapidly deteriorating patient’. This could suggest change to learning experiences.
- data related to learner confidence about their performance and knowledge ( Menon *et al*., 2017) on assessment items is generated. The areas where learner confidence and expected performance misaligns for groups of learners could suggest change to learning experiences.
- numerical and qualitative data is aggregated to inform the decisions of competence committees ( Carney *et al*., 2023). Cohort performance data on the professionalism domain could suggest change to learning experiences in the clinical setting.
- analysing aggregated, larger scale data from Workplace Based Assessment for the presence of actionable feedback to learners. Sparse actionable feedback suggests change to the briefing of clinical supervisors about how to give feedback to learners.

However, these affordances must look beyond provision of technology software and hardware and ensure support at the level of individual learners and faculty. Both groups need instruction in the use of technology to learn, assess, improve and progress, with a greater focus on the quantified self ( Malecka & Boud, 2021). An increased focus on co-creation and shared design enables greater engagement, confidence in the learning/assessment technology and authentic design, aligned to workplace tasks ( Cumbo & Selwyn, 2022; Divami, 2021). Technology provides powerful affordances to deliver interventions to support learning, including:

- At the level of the learning/assessment environment, e.g. using augmented reality to support just in time learning and assessment during placement rotations or a move to a new workplace, drawing on the theories of transition and critically intensive learning periods ( Kilminster *et al*., 2011).
- Through the support of individual cohorts or particular subgroups who may be in need of extra support, where technology can deliver targeted resources ( Foster & Siddle, 2020).
- At the level of the individual, where technology mediated behaviour change (e.g., incentivisation, nudging) can support learning, assessment and actioning feedback, including the use of remote mentoring and coaching ( Damgaard & Nielsen, 2018).

Learning, assessment and technology are increasingly intertwined (and interdependent) and building integrated coherence between these three areas is a critical goal.

### 2) Steps towards shared responsibilities for equity, diversity, inclusion and wellbeing

A key aspect for validity is attention to the responsibilities for ensuring equity, diversity, inclusion and wellbeing, when designing, implementing and evaluating assessments and the impact on learning. Much work has been situated around debates on ‘decolonisation’ and what this means in higher education contexts ( Bhambra *et al*., 2018) and within the realm of assessment the focus has been on attainment gaps between students from different backgrounds and whether these are likely to be overcome within various different education systems ( Holmwood, 2018). When the ultimate aim of all health professions education is to strive to improve patient care, to compete over practice in this area feels at odds with the ethos of what we wish to achieve, and the pandemic showed us that despite years of working in competition, we can work in a more agile way, across borders both nationally and internationally ( Fuller *et al*., 2020). One of the challenges for educators is that many of us are only at the start of the journey in terms of acknowledging these responsibilities and figuring out how we can ensure that we view our curriculum, teaching-learning and assessment activities through the lenses of equity, diversity, inclusion and wellbeing.

In both the Performance Assessment and Technology Enhanced Assessment consensus statements ( Boursicot *et al*., 2021; Fuller *et al*., 2022), there were themes around the responsibilities that we, as educators, have to share best practice globally and these responsibilities further prompt key questions:

a) How might we think about reducing inequity in assessment practice?
b) How do we approach embracing diversity of assessors involved in assessments?
c) How can we ensure appropriate inclusion of voices for their viewpoints and perspectives: students, patients, other stakeholder groups?

Technology offers affordances for each of these. For reducing inequity in assessment practice, we can use technologies to bring multi-jurisdiction groups together to learn from and partner with educators across different settings. A longitudinal approach to supporting each other will create opportunities to share learning and is a social responsibility ( Fuller *et al*., 2022). Within the realm of equality related to social justice, we all have increasing social responsibility to use less, and that includes technological resources. Many of us could do what we need with what we already have, and so thinking creatively with technology rather than constantly upgrading will help both equality and sustainability in the longer term. New technology involves hidden costs, including faculty and student training. Doing better with what we already have can therefore be both more efficient, but also help us share best practice and potentially reduce inequity if we are sharing this practice more widely.

For embracing diversity, the performance assessment consensus statement highlighted the evidence that has supported the value of rater (assessor) variance as meaningful ( Boursicot *et al*., 2021). Rather than attempting to avoid or control differences between assessors, these differences should be embraced and actively designed into the process and taking a longitudinal, holistic view is important in relation to inclusivity (ibid). To enact this, we need diversity within our assessor groups, and a way to understand this diversity in places that we do not always see; the diversity of examiners across an OSCE for example can be influenced, but we may not know who assessors of learners are in the clinical practice setting. Alongside this, we need to ensure any data interpretation is holistic, and ensure we are not disadvantaging certain groups through our assessments, either in their design, in our interpretation of results, or in the use of technology to perform the same function ( Broussard *et al*., 2018).

The work on the assessment life cycle ( Fuller *et al*., 2022) reminds us that we need to think about not just protecting from discrimination, but actively including voices from a range of backgrounds and experiences. We so often see equity, diversity and inclusivity mentioned without acknowledgement that what happens when we get those things right – that is, we create wellbeing for our staff, students and patients. When individuals feel they are represented, when they have a voice, and that we can assure ourselves there is as much fairness and equity as is possible in a diverse world, it follows that much of the anxiety in terms of learning and assessment – in terms of what feels “unfair” – is addressed and results in improved wellbeing for both learners and the educators.

### 3) Steps towards achieving a sustainable assessment culture

A key aspect of validity links to the purposes of assessments. We regularly focus on the learning purposes of assessment, that is, assessment should help learners improve. Some assessments are designed with a different purpose: to make judgements of clinical competence or other attainment. The limitations of single assessment tasks to meet multiple purposes has been noted by Norcini *et al*. (2018) who highlight a need for carefully designing the system of assessment and that this system must be more than a collection of assessments. The purpose of making a high stakes judgement of performance to assure competence of health professionals, and thus fulfil our collective duties to patient safety, the public, the profession, and learners, is one distinct purpose. This purpose must, in a system of assessment, coexist with other key purposes such as supporting and evidencing longitudinal learner development.

High quality feedback to learners to inform their learning, and sustain that learning across time, means that this is an important shift. We need to attend to feedback not only from single assessments, but also to feedback within the system of assessment, and this is needed from a longitudinal perspective. Whilst many assessment taxonomies only describe hierarchies of cognitive systems (e.g., comprehension, analysis, synthesis), work by Marzano and Kendall highlights the importance of both metacognitive and self-systems within assessment ( Marzano & Kendall, 2007). Namely, it requires us to consider not just the assessment/task, but the learner’s reaction to it (and impact on engagement, motivation and goal setting). This has important consequences for the design, delivery, timing and consequences of assessment, particularly if assessment is scheduled during busy teaching periods/clinical placement activities.

Sustainable assessment ( Boud & Soler, 2016) has emerged from research and practices that focus on assessment *for* learning. By reviewing many of the unintended consequence of (high stakes) assessment, the concept of sustainable assessment provides a framework for more holistic practice, allowing the purposeful design of assessment encounters that continue to positively shape approaches to learning and development be sustained beyond any given assessment. By reintegrating assessment of competency with active learner development theories (e.g., growth mindset) and the environment in which learning (and remediation) take place, it provides a powerful transformative approach.

Key questions we ask to understand how feedback positively shapes approaches to learning and performance across time, beyond any given assessment, and with technology support, include:

a) How might we use theories of sustainable assessment to reshape a comprehensive programme of assessment to support both a learner’s current and future learning needs?
b) How are we able to integrate assessment of competency, with knowledge about active learner development?
c) How do we use assessment outcomes to further support learner development and wellbeing initiatives?

In both the Performance Assessment and Technology Enhanced Assessment consensus statements, we touched on consequences of assessment design on learner responses to our systems of assessment. Technologies such as assessment management systems and ePortfolios enable us to better analyse and understand the consequences of our assessment design. For example, technology enables instant visibility when learners have patterns of engagement in Workplace Based Assessments that correlate strongly with poor performance, prompting early intervention such as ‘nudges’ ( Thaler & Sunstein, 2008).

Active learner development work has generated an understanding of the importance of a ‘growth mindset’ ( Dweck, 2012) on how learners approach challenging learning and performance. As technology designers may not always account for active learning strategies, educators and students can co-design feedback reports that are generated efficiently by technology and also reinforce active learning strategies. Examples include engaging students in understanding:

- the importance of feedback framed as action by the learner,
- the minimal value (and often negative impact) for learning of information that compares learners with others, and,
- the importance of self-monitoring assessment data and engaging in goal setting (within or outside the system).

### 4) Steps towards balancing resources in global contexts

A key aspect of validity is the context in which learners complete assessments, and this includes the resources available within the education system, reflecting a community and societal resourcing context. Health professions education in a range of countries share a common goal of educating safe and effective clinical practitioners. However, the learning experiences, assessment design and other components of education reflect the technological resources available. Key questions which educators in countries may differ in their responses include:

1. How do we prioritise resources, particularly technological resources, for assessment and learning?
2. How do we develop best practices which account for our specific needs and our technological context?
3. How do we contribute to global literature to provide a balanced and realistic account of approaches and practices?

Different countries and jurisdictions reflect the history, needs and responses to the societal context. Increasingly we recognise the impact of colonisation on First Nations peoples, inter-generational disadvantage, and the effect of poverty. Technology for learning and assessment that is expensive; requires significant training and/or personnel; is misaligned with learner technology background or experience; or does not reflect technologies in local educational or clinical settings, is unlikely to be feasible. Academic work is largely drawn from technologically well-resourced areas and this knowledge may not always be helpful for other jurisdictions. Thus, the knowledge base in the current literature does not represent all the global stakeholders in the field of medical and health professional education. The first step towards supporting validity of our assessments from a global resources perspective is to generate multiple avenues for learning together and create a knowledge base relevant to contexts with different access to resources. This collective effort needs also to focus beyond resource and technology and examine the importance of culture, e.g., through the lens of improvement and what we can share from frugal innovation ( Hossain, 2017).

## Conclusion

The steps outlined above are suggested ways to consider learning, assessment and technology. Each of the four steps that we addressed: importance of integration that is coherent; sharing responsibilities for equity, diversity, inclusion and wellbeing; sustainability; and balancing resources in global contexts, represents progress in our ability to support validity of our assessment practice and enhance learning.

We argue that all involved in learning and assessment in health professional education, need to ensure strong alignment between theoretical understandings from the fields of learning sciences and assessment, in order to evaluate how to use, implement, and adapt understandings from the field of technology. This is critical when designing learning experiences and assessing learning and/or achievement.

Questions we need to address moving forward include: How do we leverage technology to analyse alignment, from the learner/cohort perspectives? How do we look for evidence of success with practical strategies we adopt for supporting equity, diversity, inclusivity and wellbeing? How do we investigate the consequences of the design of our assessments, using technology? How do we work together with global partners, to design resource prioritisation fit for context?

Progress towards understanding our responses to these questions supports validation for contextualised applications of technology for assessment, and for learning. To promote this, we call for scholarship, and scholarly output, to ‘advance conversations’ ( Boud & Soler, 2016), particularly where fast evolving applications of technology connect with learning and assessment. A focus on scholarship (as well as research) will promote active collaboration across different settings and strengthen diversity, as well as enable rapid dissemination of knowledge to inform the complexity of contemporary education of all health professionals. 

## Data Availability

No data are associated with this article.

## References

[ref-1] American Educational Research Association, American Psychological Association, & National Council on Measurement in Education: Standards for educational and psychological testing. American Educational Research Association,2014. Reference Source

[ref-2] Bhambra GK Gebrial D Nişancıoğlu K : *Decolonising the university* (1st ed.).Pluto Press,2018. Reference Source

[ref-3] Biggs JB Tang CSk : *Teaching for quality learning at university: what the student does* (4th edition. ed.).McGraw-Hill/Society for Research into Higher Education,2011. Reference Source

[ref-4] Black P Harrison C Lee C : Assessment for Learning: putting it into practice. McGraw-Hill Education,2007. Reference Source

[ref-5] Boud D : Reframing assessment as if learning were important.Routledge,2007;24–36. 10.4324/9780203964309-8

[ref-6] Boud D Soler R : Sustainable assessment revisited. *Assess Eval High Educ.* 2016;41(3):400–413. 10.1080/02602938.2015.1018133

[ref-7] Boursicot K Kemp S Wilkinson T : Performance assessment: Consensus statement and recommendations from the 2020 Ottawa Conference. *Med Teach.* 2021;43(1):58–67. 10.1080/0142159X.2020.1830052 33054524

[ref-8] Broussard KA Warner RH Pope ARD : Too Many Boxes, or Not Enough? Preferences for How We Ask About Gender in Cisgender, LGB and Gender-Diverse Samples. *Sex roles.* 2018;78(9–10):606–624. 10.1007/s11199-017-0823-2

[ref-9] Carney PA Sebok-Syer SS Pusic MV : Using learning analytics in clinical competency committees: Increasing the impact of competency-based medical education. *Med Educ Online.* 2023;28(1):2178913. 10.1080/10872981.2023.2178913 36821373 PMC9970252

[ref-10] Clauser BE Bunch MB : The history of educational measurement: key advancements in theory, policy, and practice. *Research and Perspectives.* Routledge,2022.

[ref-11] Cook DA Brydges R Ginsburg S : A contemporary approach to validity arguments: a practical guide to Kane's framework. *Med Educ.* 2015;49(6):560–575. 10.1111/medu.12678 25989405

[ref-12] Cotton DRE Cotton PA Shipway JR : Chatting and cheating: Ensuring academic integrity in the era of ChatGPT. *Innov Educ Teach Int.* ahead-of-print (ahead-of-print),2023;1–12. 10.1080/14703297.2023.2190148

[ref-13] Cumbo B Selwyn N : Using participatory design approaches in educational research. *Int J Res Method Educ.* 2022;45(1):60–72. 10.1080/1743727X.2021.1902981

[ref-14] Damgaard MT Nielsen HS : Nudging in education. *Econ Educ Rev.* 2018;64:313–342. 10.1016/j.econedurev.2018.03.008

[ref-15] Divami : Top 5 principles of EdTech design. 2021; [accessed 2023 May 21]. Reference Source

[ref-16] Dweck CS : Mindset.Robinson,2012.

[ref-17] Foster E Siddle R : The effectiveness of learning analytics for identifying at-risk students in higher education. *Assess Eval High Educ.* 2020;45(6):842–854. 10.1080/02602938.2019.1682118

[ref-18] Fuller R Goddard VCT Nadarajah VD : Technology enhanced assessment: Ottawa consensus statement and recommendations. *Med Teach.* 2022;44(8):836–850. 10.1080/0142159X.2022.2083489 35771684

[ref-19] Fuller R Joynes V Cooper J : Could COVID-19 be our 'There is no alternative' (TINA) opportunity to enhance assessment? *Med Teach.* 2020;42(7):781–786. 10.1080/0142159X.2020.1779206 32552191

[ref-20] Holmwood J : Inegalitarian populism and the university: British reflections on Newfield's *The Great Mistake: How We Wrecked Public Universities and How We Can Fix Them*: Book review symposium. *Br J Sociol.* 2018;69(2):510–517. 10.1111/1468-4446.12339_5

[ref-21] Hossain M : Mapping the frugal innovation phenomenon. *Technology in society.* 2017;51:199–208. 10.1016/j.techsoc.2017.09.006

[ref-22] Issenberg SB : Ottawa 2010 Conference - Consensus Statements and Recommendations. *Med Teach.* 2011;33(3):181–2. 10.3109/0142159X.2011.551562 21345057

[ref-23] Kane MT : Validating the Interpretations and Uses of Test Scores. *J Educ Meas.* 2013;50(1):1–73. 10.1111/jedm.12000

[ref-24] Kilminster S Zukas M Quinton N : Preparedness is not enough: understanding transitions as critically intensive learning periods. *Med Educ.* 2011;45(10):1006–15. 10.1111/j.1365-2923.2011.04048.x 21916940

[ref-25] Malecka B Boud D : Fostering student motivation and engagement with feedback through ipsative processes. *Teaching in Higher Education.* head-of-print (ahead-of-print),2021;1–16. 10.1080/13562517.2021.1928061

[ref-26] Marzano RJ Kendall JS : The new taxonomy of educational objectives.2nd ed. California (US): Corwin Press,2007. Reference Source

[ref-27] Menon A Gaglani S Haynes MR : Using "big data" to guide implementation of a web and mobile adaptive learning platform for medical students. *Med Teach.* 2017;39(9):975–980. 10.1080/0142159X.2017.1324949 28510500

[ref-28] National Academies of Sciences Engineering & Medicine: How People Learn II: Learners, Contexts, and Cultures.The National Academies Press,2018;346. Reference Source

[ref-29] Norcini J Anderson MB Bollela V : 2018 Consensus framework for good assessment. *Med Teach.* 2018;40(11):1102–1109. 10.1080/0142159X.2018.1500016 30299187

[ref-30] Piaget J : The psychology of intelligence.Routledge & Paul,1950.

[ref-31] Shankar PR : Artificial intelligence in health professions education. *Archives of Medicine and Health Sciences.* 2022;10(2):256–261. 10.4103/amhs.amhs_234_22

[ref-32] Thaler RH Sunstein CR : Nudge: Improving decisions about health, wealth, and happiness.Yale University Press,2008;293. Reference Source

[ref-33] von Glasersfeld E : Radical constructivism: a way of knowing and learning.Falmer Press,1995;213. Reference Source

[ref-34] Wertsch JV : Vygotsky and the social formation of mind.Harvard University Press,1985;262. Reference Source

[ref-35] Wiliam D : Embedded formative assessment (Second edition. ed.).Solution Tree Press,2018.

