# Supplementary material for: Supporting validity: steps to contextualise applications for technology and assessment, for learning
Source: MedEdPublish (2016). 2023 Jul 19;13:37. [Version 1] doi: 10.12688/mep.19688.1 (PMC10587818; doi:10.12688/mep.19688.1)
Supplement: Peer review history

# Reviewer report: Reviewer response for version 1

**DOI:** 10.21956/mep.21089.r34807

**Published:** 2023-10-06

**Authors:** Helen Wozniak, Christine Devine

**Affiliations:** 1 Academy for Medical Education, The University of Queensland, Saint Lucia, Queensland, Australia

In this article Kemp and colleagues are challenging us to think carefully about the interconnected nature of technology, learning and assessment. They do this by focusing our attention on key areas for consideration as we are drawn into these voluminous medical education spaces. Drawing on evidence-informed best practice from two Ottawa consensus statements, they unpack four key aspects of the assessment validity framework (coherence, equity, diversity, inclusion and wellbeing, sustainability, global resources).

 While we agree with Harden (2023) that the article does take the reader time to distil and consider its key messages, this is partially due to the mystique that surrounds the concept of validity and assessment.  While table 1 summarises the key messages from the two consensus statements, the article provides a rich array of further examples to explain each validity component, suggesting areas for contemplation that can be applied to any assessment format, not just performance-based assessment. For each aspect they identify three questions that extend the principles and synthesized evidence outlined in these consensus statement. Readers will gain the most value from this paper when they examine their specific context in light of these key questions.  

 In considering the interdependent nature of technology, learning and assessment, a common theme that runs across all four aspects of the validity framework is the value of developing self-efficacy and learner-centred approaches to achieve positive impact.  When coherence is realised, how might we use the data to shift the focus – micro (individual or course) to macro (cohort or program) – to achieve an equitable learning experience for all? The call to action by the authors to share scholarship in this space offers both a sustainable and scalable option for transformative learning for health professions educators across contexts.

 As noted by Thomas and Ellaway (2021) making decisions in health professions education is context-bound requiring careful consideration of local factors such as resourcing, the culture of the organisation and the values of those within it. So how can the emerging evidence from consensus statements be translated into local policy and practice initiatives? This contribution hones our thinking, illuminates important considerations and provide us with a way forward.

*Is the topic of the opinion article discussed accurately in the context of the current literature?*

 Yes. An area that could be clarified to the reader is the purpose and structure of Table 1. This table summarises the key messages from the consensus statements, but it is unclear if the rows have a common theme across each row for the two consensus statements listed, or is each column representing a list of key messages independently? If the former is the intent, perhaps a sub heading for each row would assist to improve clarity to connect each column. The table is only referred to once at the beginning of the paper, should additional references be made throughout the opinion piece as a reminder of the relationship the validity components as they are discussed and these key messages?

*Are all factual statements correct and adequately supported by citations?*

 Yes the additional reference provides a good stepping off for the reader to explore further background and educational principles.

*Are arguments sufficiently supported by evidence from the published literature?*

 Yes it was pleasing to connect the four key aspects of the validity framework with the broader educational literature beyond a focus on medical education. The importance of interdisciplinarity in educational research is also highlighted by Wainwright et al. (2020).

*Are the conclusions drawn balanced and justified on the basis of the presented arguments?*

 Yes

Is the topic of the opinion article discussed accurately in the context of the current literature?

Yes

Are arguments sufficiently supported by evidence from the published literature?

Yes

Are all factual statements correct and adequately supported by citations?

Yes

Are the conclusions drawn balanced and justified on the basis of the presented arguments?

Yes

Reviewer Expertise:

Assessment, workplace-based learning, educational technology, learning design, learning analytics

We confirm that we have read this submission and believe that we have an appropriate level of expertise to confirm that it is of an acceptable scientific standard.

## References

[1] : Rethinking implementation science for health professions education: A manifesto for change. *Perspect Med Educ* .2021;10(6) : 10.1007/s40037-021-00688-3 362-368 10.1007/s40037-021-00688-3 34757538PMC8633355

[2] : Why educational research should remain mindful of its position: Questions of boundaries, identity and scale. *British Educational Research Journal* .2020;46(1) : 10.1002/berj.3594 1-5 10.1002/berj.3594

# Reviewer report: Reviewer response for version 1

**DOI:** 10.21956/mep.21089.r34800

**Published:** 2023-10-05

**Authors:** Stephen Tobin

**Affiliations:** 1 School of Medicine, Western Sydney University, South Penrith, New South Wales, Australia

The authors invoke validity as a lens to explore the relationships between four intersecting aspects of learning and assessment specifically in relation to technology. This paper builds on the Ottawa papers, acknowledging overlapping authorship. The authors effectively provide health professional educators with narrative to inform systems of assessment.

 The article is important because many medical and other health professional institutions still make use of extensive manual handling of assessment data, with variable use of IT; this is unsurprising as many still do not have clear, transparent and well resourced systems of assessment.

 Coherence is fundamental and this section reminds how technology can help. Cohort data can be monitored and individual data sampled, enabling review of curricular experiences and placements, as well as proactive intervention for poor performance or lack of engagement, being the early pointers to (a) needing help and (b) likely failure at end-of- subject major examinations. Such systems should be responsive and recognise that coaching should be seen as normal: some learners will need more than others.  Technology makes human review easier, but it still requires time for this thoughtful input. Competency panels take this further to support progression.

 Equity and diversity are important. Technology costs but much can be done on smartphones.

 Diversity in many contexts is discussed, noting there is richness here for assessment, which becomes more holistic. The learner view and the patient responses can be built-in through co-development and evaluation.

 Portfolios that integrate assessments including those from the workplace allow evidence of progress, self-reflective comments as well as transparency. The intrinsic role of feedback throughout ties into this. Learner ownership of their progress (the portfolio and more) comes into this self-directed learning. Sometimes, as the reviewer has done, it’s about designing and implementing such a system, and encouraging all involved to use it for student learning.

 Finally, health professional educators can share what “works” and with technology partners develop ways that this can technology, learning and assessment - the “space” – used in many contexts. This is a valuable and well-referenced opinion article that all should consider.

Is the topic of the opinion article discussed accurately in the context of the current literature?

Yes

Are arguments sufficiently supported by evidence from the published literature?

Yes

Are all factual statements correct and adequately supported by citations?

Yes

Are the conclusions drawn balanced and justified on the basis of the presented arguments?

Yes

Reviewer Expertise:

Medical education

I confirm that I have read this submission and believe that I have an appropriate level of expertise to confirm that it is of an acceptable scientific standard.

# Reviewer report: Reviewer response for version 1

**DOI:** 10.21956/mep.21089.r34322

**Published:** 2023-08-22

**Authors:** Ronald M. Harden

**Affiliations:** 1 An International Association for Medical Education, Dundee, Scotland, UK

I recommend this insight into assessment, which provides a valuable perspective on the subject. It looks at the technology-assessment relationship.

 Frans Johansson (2006) in *The Medici Effect* argued that when you step into an intersection of fields, disciplines or cultures, you can combine existing concepts into a large number of extraordinary new ideas. Kemp et al. have made a valuable service to medical education by bringing together the recommendations from the Ottawa Consensus Statement on Technology and the Consensus Statement on Assessment.

 They have demonstrated the important relationship between performance assessment and the use of technology and shown how the integration of technology has dramatically influenced performance assessment by enhancing its precision, efficiency and scope. They suggest that the successful integration of technology, however, requires thoughtful consideration.

 This paper is not an easy read but it is well worth the time required to take on board its key messages, which are clearly argued. It represents a valuable contribution to the health professions education literature.

Is the topic of the opinion article discussed accurately in the context of the current literature?

Yes

Are arguments sufficiently supported by evidence from the published literature?

Yes

Are all factual statements correct and adequately supported by citations?

Yes

Are the conclusions drawn balanced and justified on the basis of the presented arguments?

Yes

Reviewer Expertise:

Medical education

I confirm that I have read this submission and believe that I have an appropriate level of expertise to confirm that it is of an acceptable scientific standard.

## References

[1] : The Medici Effect: Breakthrough Insights at the Intersection of Ideas, Concepts, and Cultures. *Harvard Business Review Press* .2006;
